# Experiences of Using Cochrane Systematic Reviews by Local HTA Units

**DOI:** 10.34172/ijhpm.2020.133

**Published:** 2020-08-01

**Authors:** Thomas G. Poder, Marc Rhainds, Christian A. Bellemare, Simon Deblois, Imane Hammana, Catherine Safianyk, Sylvie St-Jacques, Pierre Dagenais

**Affiliations:** ^1^Department of Management, Evaluation and Health Policy, School of Public Health, University of Montreal, Montréal, QC, Canada.; ^2^Centre de Recherche de l’Institut Universitaire en Santé Mentale de Montréal, CIUSSS de l’Est de l’Île de Montréal, Montréal, QC, Canada.; ^3^HTA Unit, CHU de Québec – Université Laval, Québec, QC, Canada.; ^4^Department of Multidisciplinary Services, Clinical Quality Division, CIUSSS de l’Estrie-CHUS, Sherbrooke, QC, Canada.; ^5^HTA Unit, CHUM, Montréal, QC, Canada.; ^6^HTA Unit, CIUSSS de la CapitaleNationale, Québec, QC, Canada.; ^7^HTA Unit, CIUSSS de l’Estrie – CHUS, Sherbrooke, QC, Canada.; ^8^Department of Medicine, Faculty of Medicine and Health Science, University of Sherbrooke, Sherbrooke, QC, Canada.

**Keywords:** Cochrane Systematic Review, Systematic Review, Health Technology Assessment, Hospital, Quebec

## Abstract

This study evaluated the use of Cochrane systematic reviews (CSRs) by Quebec’s local health technology assessment (HTA) units to promote efficiency in hospital decision-making. An online survey was conducted to examine: Characteristics of the HTA units; Knowledge about works and services from the Cochrane Collaboration; Level of satisfaction about the use of CSRs; Facilitating factors and barriers to the implementation of CSRs evidence in a local context; Suggestions to improve the use of CSRs. Data accuracy was checked by 2 independent evaluators. Ten HTA units participated. From their implementation a total of 321 HTA reports were published (49.8% included a SR). Works and services provided by the Cochrane collaboration were very well-known and HTA units were highly satisfied with CSRs (80%-100%). As regards to applicability in HTA and use of CSRs, major strengths were as follow: Useful as resource for search terms and background material; May reduce the workload (eg, brief review instead of full SR); Use to update a current review. Major weaknesses were: Limited use since no CSRs were available for many HTA projects; Difficulty to apply findings to local context; Focused only on efficacy and innocuity; Cannot be used as a substitute to a full HTA report. This study provided a unique context of assessment with a familiar group of producers, users and disseminators of CSRs in hospital setting. Since they generally used other articles from the literature or produce an original SR in complement with CSRs, this led to suggestions to improve their use of CSRs. However, the main limit for the use of CRS in local HTA will remain its lack of contextualisation. As such, this study reinforces the need to consider the notion of complementarity of experimental data informing us about causality and contextual data, allowing decision-making adapted to local issues.

## Introduction and Context

Due to the amount and the ongoing growth of the literature related to medical knowledge, systematic reviews (SRs) appear as a fundamental component in the process of producing relevant recommendations for clinicians and decision-makers.^1,2^ To support policy and practice decisions, Cochrane systematic reviews (CSRs) are one of the best known and most trusted sources of evidence-based in healthcare.^3,4^ Cochrane handbook described SR as a review that “attempts to collate all empirical evidence that fits pre-specified eligibility criteria in order to answer a specific research question. It uses explicit, systematic methods that are selected with a view to minimizing bias, thus providing more reliable findings from which conclusions can be drawn and decisions made.”^4^ However despite the fact that 96% of decision-makers reported that they valued SRs in the development of new guidelines and 60% consider SRs as very important when compared with other sources of evidence, it appears clearly that SRs are underutilized.^5^ In fact, although numerous international initiatives have been taken to support the use of SRs, variation in the uptake of the latter by decision-makers persist.^6^

We acknowledge that healthcare professionals, local policy-makers and health system managers, can face several challenges when attempting to utilize these evidences.^7^ These include the difficulties in applying global evidence in a local clinical context by adapting evidence from SRs so that it is locally relevant, and the way in which ‘use’ can be conceptualized.

 The aim of this paper is to describe how CSRs are used by local health technology assessment (HTA) units to promote efficiency in hospital decision-making. This was done by identifying facilitators and barriers to its utilisation and then by specifying what type of SRs are needed for local users. Finally, we provide elements to support a better understanding about its local applicability.

## Methods

A web-based survey was conducted between August 30 and September 10, 2013 using the Surveymonkey^®^ platform. The survey was developed by the authors of this study and was validated by an external academic expert in survey and HTA research as regards to the relevance of the items and the univocity of the sentences. All ten local HTA units implemented in Quebec since 2001 were solicited by email (ie, the person in charge of the unit). These units were members of a local HTA units’ community of practices (CoP) of the province of Quebec, Canada. The survey was anonymous but respondents were aware that some of their answers could indicate which HTA units were interviewed. Some respondents also contacted the research team to be able to respond precisely to some questions. In addition, when the results of the survey were presented to the members of at the CoP, some of the participants spontaneously identified themselves to explain some of the results (ie, open-ended questions). To ensure accuracy, data was checked by 2 independent evaluators (TGP and CAB).

 The survey included (1) Questions to describe the HTA unit (ie, date of HTA unit creation, number of members and occupation, number and type of their publications); (2) Use and knowledge of Cochrane collaboration works and services; (3) Perceptions of the respondents concerning their experience using CSRs. The ease of using and consulting the reviews, their thematic coverage, methodological characteristics and applicability in clinical settings was assessed; (4) Barriers and facilitators associated with using CSRs into HTA at the clinical level (measured with 2 open questions); (5) Satisfaction and experience of utilisation of CSRs (measured with 10 characteristics of the format of CSRs and open-ended questions). Questions about the use and satisfaction of CSRs were rated on a 4-point Likert scale (strongly agree/satisfied, somewhat agree/satisfied, somewhat disagree/unsatisfied, strongly disagree/unsatisfied). In the final section, respondents were asked to write suggestions to improve the use of CSRs in their own context.

## Results

###  Characteristics of the Health Technology Assessment Units

The response rate was 100%. Eight of the ten HTA units were from university hospital centers and institutes and 2 other were from health and social services centers. All units were created between 2001 and 2010, including 40% in the last 2 years. Only one unit over 10 had access to a Cochrane representative in their health facility. The average team size was 6.3 [range 2-18] people (not full-time equivalent) and 69.8% were office researchers. Other members were managers or administrative staff (23.8%), librarians (3.2%), physicians or students (3.2%). HTA units had a staff of 5 people or less in 60% of case. Furthermore, most of the staff in 40% of these units was part-time. In average, the HTA units annually produced 4.7 reports [range 2-7.3] over the last 3 years, including 4.1 published reports [range 0-7.3] and 0.6 unpublished reports [range 0-3]. Half of the units published all their reports. Table 1 provides the nature of these reports.

**Table 1:** Nature of Reports Produced Since the Creation of the HTA Units

**Nature of Reports**	**Published Reports (n = 321)**	**Unpublished Reports (n = 36)**	**All Reports (N = 357)**
SRs	49.8%	22.2%	47.1%
Narrative reviews	3.8%	2.8%	3.6%
Mini HTA	16.8%	30.5%	18.2%
Field evaluations	24.9%	27.8%	25.2%
Others	4.7%	16.7%	5.9%
Total	100.0%	100.0%	100.0%

Abbreviations: HTA, health technology assessment; SRs, systematic reviews. Notes: Systematic reviews and narrative reviews refer to full HTA including the analysis of empirical data along with an analysis of the local context; Mini HTA does not have the same definition from a local HTA unit to another but corresponds to a report that assessed only some aspects of the object under analysis; A field evaluation refers to a report that collected primary data due to a lack of evidence in the scientific literature.

###  Use and Knowledge of Cochrane Collaboration Works and Services

Almost all HTA units were familiar with CSRs (90%) (ie, one unit indicated that about half of its team was not familiar with CSRs), a large majority was aware of the existence of Cochrane meetings (80%), less known was the existence of Cochrane webinars and trainings (50%). All HTA units used CSRs in their practice, half often or always, the other half sometimes. The CSRs were used at the time of scoping and planning, and during the conduct of the HTA project (Table 2). Only one unit had participated in a Cochrane review. HTA units used CSRs in several areas, but the most common were healthcare technologies, pain, laboratory analysis, cancer, surgery and pharmacy. During the last 3 years, over 85 searches performed in the Cochrane library database for CSRs, only 21 searches were successful (24.7%). The main topics searched without success were related to pain, health economics, management, diagnostic imaging, laboratory analysis, neurology, and pharmacology.

**Table 2:** Use of the Cochrane Database to Find an Systematic Review

	**Proportion of Projects **		
	**All Projects**	**At Least Half of Projects**	**Less Than Half**
Step achievement of project			
Scoping and planning (n = 9)	67%	33%	
Production (n = 10)	60%	30%	10%

###  Perception Concerning Experience Using Cochrane Systematic Reviews 

**Figure 1:**
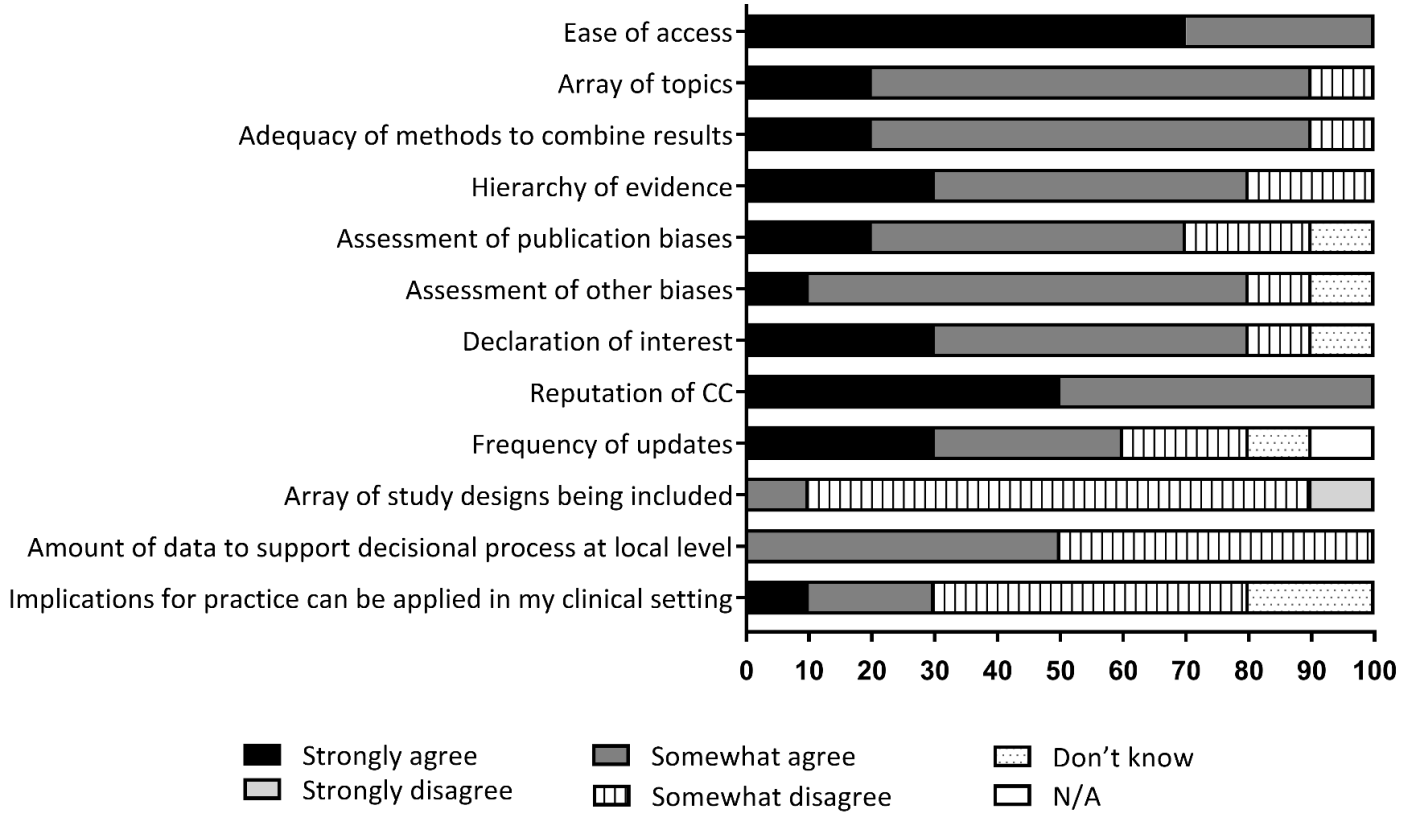
Perception of Respondents Towards Finding CSRs and Their Quality/Applicability. Abbreviations: CSRs, Cochrane Systematic Reviews; CC, Cochrane Collaboration.

The perceptions of respondents towards CSRs were generally positive (Figure 1). CSRs were deemed easily accessible through the various databases and their thematic coverage was generally considered appropriate. All respondents agreed on the importance of consulting the CSRs due the known rigorous methodology and the solid reputation of the Cochrane collaboration. The majority of respondents believed that the methods used to aggregate the various results extracted from the literature, the hierarchy of evidence found, and the evaluation of the risk of publication bias and other biases, were relevant to HTA in a healthcare setting.

However, almost all of the respondents felt that the array of study designs being included in CSRs was too narrow to meet their needs. Moreover, many respondents disagreed that CSRs contained enough data to support the decisional process and clinical practice in their setting. Also, half of the respondents disagreed with the statement that implications for practice, as described into the reviews’ conclusions, can be applied in their clinical settings (Figure 1).

###  Facilitators and Barriers

####  Facilitators

 The respondents identified 4 facilitators for using CSRs. The most often reported was the rigor of the methods used by the Cochrane collaboration. This methodology was deemed “rigorous,” “proven and constant,” “complete,” and “systematic.” This was followed by the clarity in the presentation of CSRs. Respondents also considered that the data presented in CSRs were “highly reliable” and the data analysis of “high quality.” Finally, CSRs were easy to find in bibliographical databases and main web-search engines (Google, Yahoo, Bing, etc).

####  Barriers

 Four barriers were identified by respondents. The first was related to the fact that CSRs tend to favor the data extracted from randomized clinical trials (RCTs), while often, the issues being analysed by the HTA units involve emerging technologies and interventions, on which none or few RCTs have been published. In this setting, one respondent noted that “Cochrane reviews are excellent. The problem is that the topics we face do not easily lend themselves to SRs utilising the Cochrane method because of a lack of evidence.” A second barrier was that some respondents did not find CSRs relevant to the themes they assess. The third barrier stated was that the data presented in CSRs are withdrawn from the context into which they were observed. One respondent noted that “the analysis gives more weight to statistical analysis than to clinical reality (eg, in the choice of indicators).” In addition, CSRs collected data from emerging countries whose contexts differ enormously from that of Quebec. Finally, respondents considered that CSRs should include studies of a larger array of methodological designs, like observational and qualitative studies, as well as grey literature. The inclusion of data from such studies would contribute to deepen the analysis pertaining to safety and clinical effectiveness and assess the implementation context of technologies and intervention modes.

###  Satisfaction and Experience Using the CSRs in HTA in a Clinical Setting

**Figure 2:**
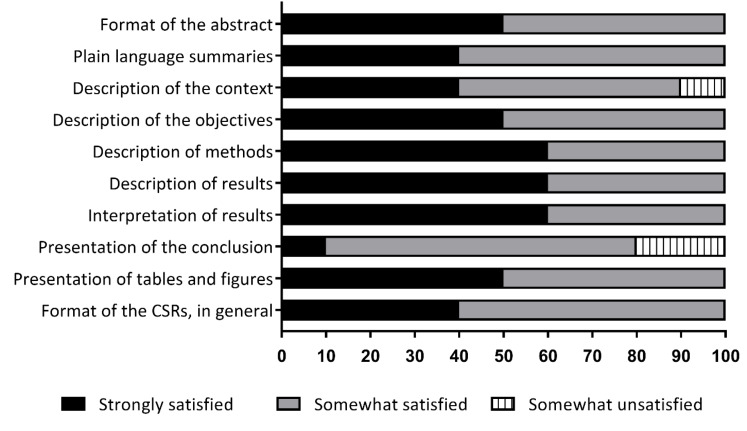
Satisfaction and Experience Using the CSR in HTA in a Clinical Setting. Abbreviations: CSR, Cochrane systematic review; HTA, health technology assessment.

The analysis of the data revealed that respondents were generally satisfied with the format of CSRs (Figure 2). The open-ended question revealed that the use of CSRs by members of their unit was an enriching, satisfying, useful experience, and that it reduced research tasks in some cases. They were enthusiastic when they found CSRs relevant to their topic, but disappointed when it was not applicable. However, some respondents were dissatisfied with the description of the context into which the interventions assessments were conducted and how the reviews themselves were carried out.

 Almost all HTA units considered that CSRs can be incorporated into work in local HTA (90%), but a large majority considered that CSRs did not prevent their unit from conducting their own SR (90%). Some specified that research needed to be expanded to include other data, that in some cases the level of evidence was insufficient and some outcomes were missing. Some units systematically use the CSRs for each evaluation project, coupled with other articles with less controlled studies. Others were especially interested in quick reviews, mapping, and scoping ones.

## Discussion

 This survey showed that CSRs have a very good reputation and were consulted by HTA unit members. However, only half of the units considered that CSRs contained enough information to help in the local decision-making process and that their conclusions may be relevant to their own clinical settings.

Dobbins et al reported that a majority of decision-makers used SRs in the development of new guidelines and consider SR as very important when compared with other forms of evidence.^8,9^ Actually, our results showed that, in Quebec, all HTA units consulted CSRs and considered that CSRs are useful and should be incorporated in their work. Two units even mentioned that the existing CSRs have avoided performing a new SR. On the other hand, a large majority considered that they still need to conduct a SR including non-RCTs, by mentioning that the level of evidence in CSRs was insufficient, with too few SR in the mental health, physical readaptation and rehabilitation area, and that they should expand the inclusion criteria to perform reviews more in line with the real world.

In this survey, the main facilitators that were reported for the use of CSRs were their methodological rigor, a clear and standardized presentation, a high reliability, and quality of analyzes. This echoes to the fact that CSRs are actually recognized in the literature for their rigorous methodology and concise summaries.^3,10-13^ Another facilitator is that CSRs were easy to find in bibliographical databases and by using main web-search engines (Google, Yahoo, Bing, etc). Wallace et al published a SR of facilitators to improve utilisation of SRs and meta-analyses.^7^ There are many facilitators, but the most common are that SR improves knowledge; a content that includes benefits, harms, and cost; training in use and peer-group support. For Dobbins et al, managers or directors use SRs more than medical and associate medical officers of health. Other predictor of use SRs were: expecting to use a SR in the future; that they overcame the barrier of limited critical appraisal skills; and perceived the reviews as being easy to use.^9^

Although SRs and CSRs are well-recognized, they are still underused.^5^ The main barriers we found in this survey were that the topics performed by HTA units often do not lend themselves well to a SR, a lack of evidence in CSRs, and that the existing CSRs were not always relevant to the themes they assess. Moreover, the data are often out of local context and do not sufficiently consider the clinical reality. More generally, CSRs assess the level of evidence associated to a causal relationship but do not consider the socio-cultural and organisational context in which health and social services interventions are conducted. This is reinforced by the fact that CSRs generally draw their evidence from RCTs. As a matter of fact, the appraisal of a larger array of methodological designs, like observational and qualitative studies, as well as grey literature, would help to deepen the analysis of safety and clinical effectiveness and assess the implementation context of technologies and intervention modes. However, these designs are deemed of lower methodological quality by the Cochrane collaboration while they are generally accepted in HTA standards.^14-18^ Moreover, the inclusion of qualitative studies would help strengthen the appraisal of the implementation of technologies and intervention modes as well as its context.^19^ Lavis et al^11^ also made this remark that SRs on other types of studies like qualitative or social services studies are less frequent.^20-23^ Later he reported that policy-makers and stakeholders need many types of SRs, reviews of observational studies, of qualitative studies, of effectiveness studies, reviews of economic evaluations, etc. and that the number of these reviews is increasing.^12^ All this echoes the debate surrounding the paradigm approach chosen by the Cochrane collaboration in favor of experimental designs and particularly RCTs.^24^ Considering that decision-makers cannot simply make a choice based on experimental data, but also need data about the context, experiential knowledge, values and preferences of users and professionals,^2^ this may explain why CSRs have a limited impact on clinical practice and the organization and delivery of healthcare services.^25^

Limitations of this survey include the limited number of respondents and the generalizability of the results outside Quebec. However, at the time of the survey, there were only ten HTA units implemented in Quebec’s hospitals and social services centres. As all respondents answered the survey, this limit became a strength by providing the opinion of the entire population considered (ie, internal validity). The limited amount of data allowed us to do descriptive analyzes and if these results cannot be generalized outside the province of Quebec, they provide a good idea of the use of CSRs by local HTA units. Another limitation is that the survey was conducted 6 years ago. At the time of the investigation, the CoP was essentially composed of HTA units in physical health, but since, new social service units were created. As there are very little CSRs in this area, probably these units would have been even more disappointed. Medical science produces the majority of the literature for CSRs, but in the social sciences, the use of meta-analysis is rapidly increasing.^26^ If the Cochrane collaboration is primarily associated with healthcare studies, its international equivalent for social care (as well as education, crime and justice) is the Campbell Collaboration.^27^ However, despite an increase in SRs in social care in the last decade, the number remains limited.^27,28^

As a result, a few suggestions to improve the use of CSRs by the HTA units would be: (1) To include non RCT; (2) To consider additional factors in the appraisal (eg, clinical heterogeneity, economics, organizational impacts, ethics); (3) To extend topics on social services and emerging technologies; (4) To develop the understanding about the applicability of CSRs to inform decision-making. If not, the future of CSRs in the HTA environment could be to promote best practices by summarizing the evidence only, or to be used as other sources of information in HTA report.^29^ However, since this survey was conducted, there have been several echoes from the Cochrane collaboration saying that they would make an effort to include other types of studies than randomised control trial.^30^ That’s a good start, even if the number of CSRs using qualitative and mixed methods studies is still very low and that contextualisation and transferability of evidence will remain an important challenge.^31^

## Conclusion

To our knowledge, this is the first study that evaluated the use of CSRs by local HTA units to promote efficiency in health decision-making. The results indicate that HTA units use and consult CSRs in their work, but that they generally complement it with other articles in the literature or produce an original SR according to their specific objectives. In some cases these units even produced primary data and collaborated with researchers to cope with the lack of evidence in the scientific literature.^32-35^ Many respondents supported that CSRs contained not enough data to support the decisional process and clinical practice. As a future avenue, it would be interesting to see if the opinion of the HTA units about the use of the CSRs has changed over the last 6 years.

## Acknowledgements

 We would like to thank all members of the HTA community of practice in Quebec for their participation in the study, as well as Julie Dussault, Alison Sinclair, Marie-Pascale Pomey, and Nathalie Carrier for their valuable comments. TGP is member of the FRQS-funded Centre de recherche de l’IUSMM. TGP is fellow of the FRQS.

## Ethical issues

 In accordance with the local ethics committee of participating organizations and the main policies governing research ethics in Health and Social Services in Québec, Canada, it was deemed not necessary to obtain an ethics approval for the project since: (1) It was proposed by the CoP and unanimously accepted by all its members; (2) It was not about personal data but about the professional use of CSRs by different HTA units. Under these circumstances, it was considered that no risks were associated to this project.

## Competing interests

 Authors declare that they have no competing interests.

## Authors’ contributions

 All authors contributed to conception and design, analysis and interpretation of data, and drafting and critical revision of the manuscript. TGP, MR, CAB, CS, and SSJ contributed to acquisition of data. TGP, CAB, and SD contributed to statistical analysis. CS contributed to administrative and technical support. TGP, MR, CAB, and PD contributed to supervision of the study.

## Authors’ affiliations

^1^Department of Management, Evaluation and Health Policy, School of Public Health, University of Montreal, Montréal, QC, Canada. ^2^Centre de Recherche de l’Institut Universitaire en Santé Mentale de Montréal, CIUSSS de l’Est de l’Île de Montréal, Montréal, QC, Canada. ^3^HTA Unit, CHU de Québec – Université Laval, Québec, QC, Canada. ^4^Department of Multidisciplinary Services, Clinical Quality Division, CIUSSS de l’Estrie-CHUS, Sherbrooke, QC, Canada. ^5^HTA Unit, CHUM, Montréal, QC, Canada. ^6^HTA Unit, CIUSSS de la Capitale-Nationale, Québec, QC, Canada. ^7^HTA Unit, CIUSSS de l’Estrie – CHUS, Sherbrooke, QC, Canada. ^8^Department of Medicine, Faculty of Medicine and Health Science, University of Sherbrooke, Sherbrooke, QC, Canada.
